# Endobronchial ultrasound-guided transbronchial needle aspiration for diagnosing thoracic lesions: a retrospective cohort study

**DOI:** 10.3389/fmed.2024.1383600

**Published:** 2024-05-10

**Authors:** Huibin Liao, Miaojuan Zhu, Ru Li, DeXin Wang, Dan Xiao, Yifei Chen, Zhenshun Cheng

**Affiliations:** ^1^Department of Respiratory and Critical Care Medicine, Zhongnan Hospital of Wuhan University, Wuhan, Hubei, China; ^2^Department of Respiratory and Critical Care Medicine, Macheng Second People's Hospital, Huanggang, China; ^3^Department of Respiratory and Critical Care Medicine, Qichun County People's Hospital, Huanggang, China; ^4^Department of Respiratory and Critical Care Medicine, Xishui Hospital Affiliated to Hubei University of Science and Technology, Huanggang, China; ^5^Wuhan Research Center for Infectious Diseases and Cancer, Chinese Academy of Medical Sciences, Wuhan, China

**Keywords:** endobronchial ultrasound-guided transbronchial needle aspiration, thoracic lesions, diagnostic performance, indication, real-world

## Abstract

**Background:**

Endobronchial ultrasound-guided transbronchial needle aspiration (EBUS-TBNA) is a minimally invasive technique for biopsy of lung, peri-pulmonary tissue and lymph nodes under real-time ultrasound-guided biopsy. It is used in the diagnosis and/or staging of benign and malignant pulmonary and non-pulmonary diseases. Our study is based on a large sample size, in a diversified population which provides a representative real-world cohort for analysis.

**Methods:**

Patients who underwent EBUS-TBNA procedure between September 2019 and August 2022 were included in this retrospective study. For cases diagnosed as benign and unclassified lesions by EBUS-TBNA, the final diagnosis was determined by further invasive surgery or a combination of therapy and clinical follow-up for at least 6 months.

**Results:**

A total of 618 patients were included in the study, including 182 females (29.4%) and 436 males (70.6%). The mean age of all patients was 61.9 ± 10.5 years. These patients were successfully punctured by EBUS-TBNA to obtain pathological results. The pathological diagnosis results of EBUS-TBNA were compared with the final clinical diagnosis results as follows: 133 cases (21.5%) of benign lesions and 485 cases (78.5%) of malignant lesions were finally diagnosed. Among them, the pathological diagnosis was obtained by EBUS-TBNA in 546 patients (88.3%) (464 malignant lesions and 82 benign conditions), while EBUS-TBNA was unable to define diagnosis in 72 patients (11.6%). 20/72 non-diagnostic EBUS-TBNA were true negative. The overall diagnostic sensitivity, specificity, positive predictive value, negative predictive value, and accuracy of EBUS-TBNA were 91.3%, 100%, 100%, 27.8%, and 91.6% [95% confidence interval (CI): 89.1–93.6%], respectively. In this study, only one case had active bleeding without serious complications during the EBUS-TBNA procedure.

**Conclusion:**

Given its low invasiveness, high diagnostic accuracy, and safety, EBUS-TBNA is worth promoting in thoracic lesions.

## 1 Introduction

The chest, including the lungs and mediastinum, is a predilection site for various benign and malignant tumors. A clear pathological diagnosis of chest lesions is essential for the appropriate clinical management of these lesions. However, due to the complex anatomical location and close to the heart, lung aorta, and other important structures, the mediastinal and adjacent mediastinal lung tissue biopsy is a difficult challenge (1).

Currently, several clinical diagnostic methods are employed for thoracic lesions, encompassing computed tomography (CT), positron emission tomography (PET)-CT, traditional bronchial needle aspiration biopsy, percutaneous lung biopsy, mediastinoscopy, and thoracoscopy, among others (2–5). However, CT and PET-CT are limited in their ability to offer a pathological diagnosis of these lesions (2). While conventional transbronchial needle aspiration (c-TBNA) is generally considered a safe procedure with a low reported incidence of complications, it exhibits limited ability to directly visualize lesions (3). Percutaneous lung biopsy is primarily utilized for peripheral thoracic lesions and is not suitable for mediastinal, hilar lesions, or enlarged lymph nodes^4^. On the other hand, mediastinoscopy and thoracoscopy are considered the “gold standard” for clinically diagnosing benign and malignant mediastinal lesions. Nevertheless, their application is limited due to the need for general anesthesia, substantial trauma, higher costs, limited feasibility for repeated examinations, and increased postoperative complications (4, 5).

Endobronchial ultrasound-guided transbronchial needle aspiration (EBUS-TBNA) is a minimally invasive ultrasound-guided biopsy, which provides a solution for mediastinal lymph nodes, hilar lymph nodes, lung tumors in the area near the airway and lung apex masses near the trachea (6). Real-time ultrasound monitoring allows clinicians to carefully avoid major blood vessels and other critical sites, reducing the risk of severe bleeding and enhancing sampling accuracy. Additionally, this procedure is straightforward, relatively safe, and minimally traumatic (7, 8). EBUS-TBNA is currently employed for diagnosing unexplained mediastinal and hilar lymph node enlargement, pulmonary and mediastinal masses, as well as lymph node staging and restaging of pulmonary and extrapulmonary tumors (9, 10). Furthermore, it provides ample tissue for immunohistochemical and molecular genotyping, offering significant implications for treatment options, including chemotherapy, targeted therapy, and re-biopsies after developing drug resistance (10–12).

In China, the introduction of EBUS-TBNA occurred relatively late, resulting in a scarcity of large-scale analysis studies in the past decade. This study, characterized by its extensive sample size and diverse participant population, holds substantial relevance and clinical applicability for staging both benign and malignant pulmonary and non-pulmonary diseases. This retrospective cohort study seeks to analyze the real-world diagnostic value of EBUS-TBNA with the goal of contributing to the standardized application and widespread adoption of EBUS-TBNA.

## 2 Methods

### 2.1 Study design and patients

We retrospectively analyzed all patients who underwent EBUS-TBNA in the Department of Respiratory and Critical Care Medicine, Zhongnan Hospital of Wuhan University from September 2019 to August 2022 due to mediastinal/hilar lymphadenopathy, mediastinal masses, and pulmonary nodules/masses near the airway. The study received approval from the ethics board of Zhongnan Hospital of Wuhan University (Approval number: 2023026K), and the need for informed consent was waived due to the retrospective nature of the study.

We extracted comprehensive, clinically relevant data regarding hospitalization and follow-up periods of EBUS-TBNA from relevant hospital databases. This data encompassed details such as age, gender, smoking history, indications for EBUS-TBNA, cyto- and histo-pathological diagnoses obtained through EBUS-TBNA, the number of puncture needles used, any complications, clinical treatment decisions based on EBUS-TBNA findings, and follow-up outcomes. Exclusion criteria encompassed patients who were contraindicated for needle aspiration due to abnormal anatomical findings or vascular interposition or those for whom a definitive diagnosis was unattainable due to insufficient data, inadequate pathological diagnosis, or lack of follow-up.

### 2.2 EBUS-TBNA procedures

All patients underwent EBUS-TBNA procedures following chest-enhanced CT or PET-CT examinations. Pre-procedure routine assessments, including blood tests, comprehensive metabolic panel tests, electrocardiograms, etc., were performed, and patients with contraindications, such as critical organ lesions or coagulation disorders, were excluded. Informed consent for the EBUS-TBNA procedure was obtained. Patients refrained from eating or drinking for a minimum of 4 h before the procedure. The procedure was carried out under local anesthesia (1% lidocaine) with sedation and analgesia provided using midazolam or fentanyl. EBUS-TBNA was conducted using a convex probe (ultrasonic fiber optic electronic bronchoscope BF TYPE UC260FW, Olympus), a specialized ultrasonic processor (EU-ME2, Olympus), and a 22-gauge specialized puncture needle (Vizishot NA-201SX-4022, Olympus). Prior to the procedure, conventional electronic bronchoscopy was employed for intraluminal exploration. All procedures were performed by experienced physicians from the Department of Respiratory Medicine. The operator determined the location and number of lymph nodes to be sampled, as well as the number of punctures per lymph node. Complications, including cough, hemoptysis, hypoxemia, arrhythmia, pneumothorax, mediastinal infection, and mediastinal abscess, were recorded.

### 2.3 Processing of puncture specimens

After sampling, a cell block was initially obtained by using the internal stylet to release the core from the needle. It was then blotted on filter paper to remove excess blood and immediately fixed in 10% formalin solution. Furthermore, the contents of the needle were placed on a glass slide and quickly fixed in a 95% alcohol solution. The specimens were promptly sent to the pathology department for cytological and histopathological examinations.

### 2.4 Study definitions

Cyto- and histo-pathological results of EBUS-TBNA were classified as malignant (if they contained malignant cells or atypical cells highly suspicious for malignancy), benign (if no malignant cells were observed but granulomas or other definitive benign lesion types were present), or unclassified (if the pathological findings were inconclusive, such as such as only mature lymphocytes, no malignant cells or caseous lesions). Patients diagnosed as benign or unclassified through EBUS-TBNA subsequently underwent other invasive procedures (percutaneous lung biopsy, mediastinoscopy, thoracoscopy, etc.) or received empirical therapy based on clinical diagnosis. A clinical and radiological follow-up of at least 6 months was considered for the clinical final diagnosis.

EBUS-TBNA results with a definitive benign or malignant diagnosis (e.g., lung cancer, tuberculosis, sarcoidosis, lymphoma, extrapulmonary metastatic carcinoma, etc.) were considered positive, and unclassified diagnoses were classified as negative. The diagnosis of 'reactive lymphadenopathy' was established only when pathological findings and at least 6-month follow-up did not lead to an alternative diagnosis, and it was considered a true negative. False negatives were defined the cases in which the supplemental surgical procedures lead to the change of the initial diagnosis obtained by EBUS-TBNA or when clinical progression occurred during the follow-up period.

The indications for EBUS-TBNA were reviewed and categorized into three major groups: (1) diagnosis and/or staging of thoracic space-occupying lesions (including pulmonary, mediastinal, tracheal, and esophageal space-occupying lesions with or without lymph node enlargement); (2) isolated mediastinal and/or hilar lymphadenopathy (IMHL); and (3) suspected intrathoracic recurrence of previous malignancies.

### 2.5 Statistical methods of analysis

Data analysis was carried out using IBM SPSS 22 statistical software. All data were subjected to tests for normal distribution and homogeneity of variance. Descriptive analysis was conducted, with measurement data conforming to normal distribution expressed as mean ± standard deviation (x ± s). Measurement data with skewness distribution were expressed as median values, along with maximum and minimum values. Counting data were presented as frequencies and percentages (%). Sensitivity, specificity, positive predictive value (PPV), negative predictive value (NPV), and accuracy were calculated based on the diagnosis obtained through EBUS-TBNA and the clinical final diagnosis. Receiver operating characteristic (ROC) curves were plotted, and the area under the curve (AUC) was used to assess the diagnostic value of EBUS-TBNA.

## 3 Results

### 3.1 Patient characteristics

**Figure 1:**
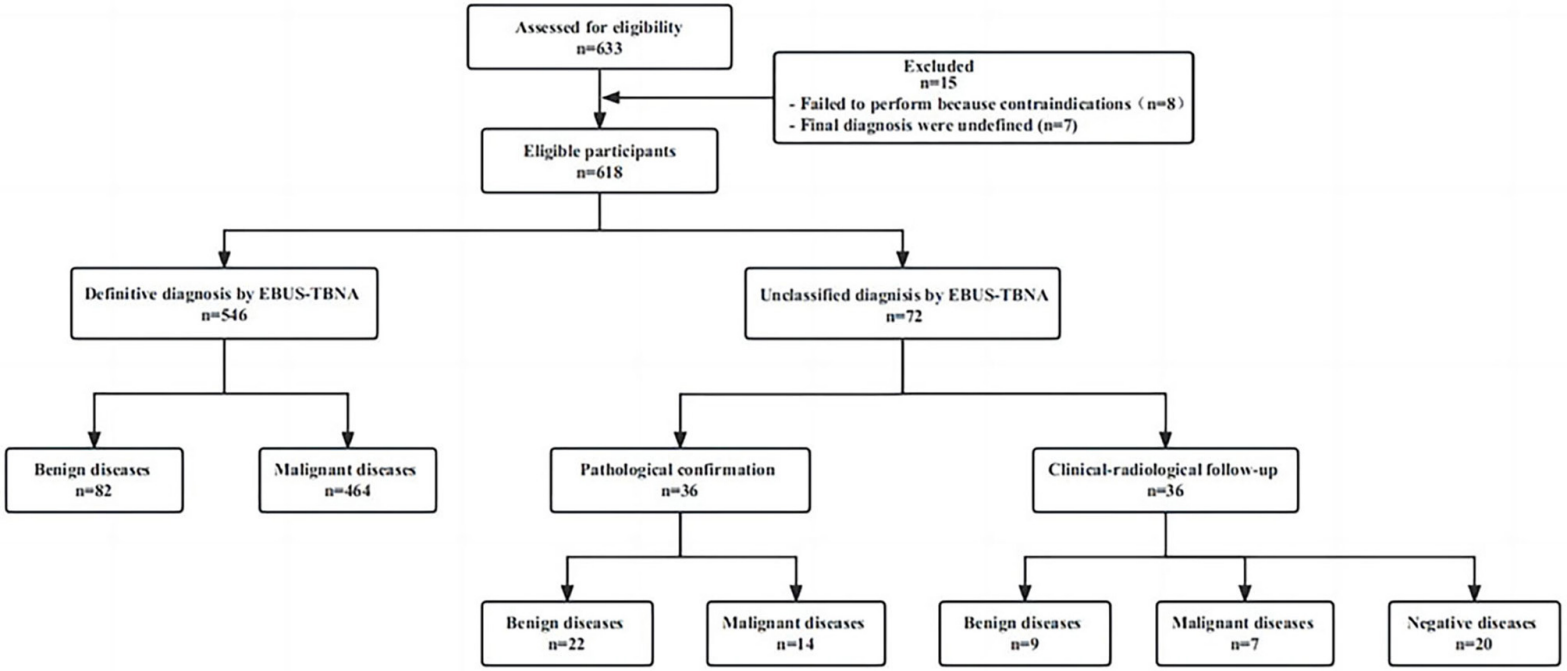
Distribution of patients according to EBUS-TBNA results and final diagnosis. EBUS-TBNA, Endobronchial ultrasound-guided transbronchial needle aspiration.

A total of 633 patients underwent initial evaluation for this study, and ultimately, 618 patients who underwent EBUS-TBNA were included and analyzed (Figure 1). The mean age of the participants was 61.9 ± 10.5 years. Among them, there were 182 females (29.4%) with an average age of 59.7 ± 11.6 years (range: 22–85 years) and 436 males (70.6%) with an average age of 62.8 ± 9.9 years (range: 18–89 years). All these patients were successfully sampled to obtain pathological results, and their characteristics and indications are summarized in Table 1.

**Table 1:** Patient characteristics and indications for EBUS-TBNA.

**Characteristic**	**Patients (*n =* 618)**
Average age (years)	61.9 ± 10.5
**Gender**, ***n*** **(%)**	
Male	436 (70.6%)
Female	182 (29.4%)
**Smoking**, ***n*** **(%)**	
Current	159 (25.7%)
Ceased	77 (12.5%)
Never	382 (61.8%)
**Indications for EBUS-TBNA**, ***n*** **(%)**	
Diagnosis and/or staging of thoracic space-occupying lesions	482 (78.0%)
IMHL	69 (11.2%)
Suspected intrathoracic recurrence of previous malignancies	67 (10.8%)

IMHL, isolated mediastinal and/or hilar lymphadenopathy.

### 3.2 Pathological diagnosis of EBUS-TBNA and clinical final diagnosis

Among the 618 patients according to the gold standard, 133 (21.5%) were benign lesions and 485 (78.5%) were malignant lesions. Among the 618 patients, EBUS-TBNA initially diagnosed 82 benign lesions (13.3%), 464 malignant lesions (75.1%), and 72 unclassified lesions (11.6%).

Among patients with unclassified lesions (*n* = 72), 19 underwent mediastinoscopy or thoracoscopic surgery, 14 underwent percutaneous lung biopsy, and three underwent extrapulmonary lymph node biopsy, leading to pathological diagnoses for 36 patients. This included 14 cases of malignant lesions and 22 cases of benign lesions. The remaining 36 patients refused to undergo further surgery. These patients underwent empirical treatment based on clinical diagnosis and received at least 6 months of clinical and radiological follow-up. The final clinical diagnosis of eight patients with initially diagnosed suspected lung cancer was seven cases of malignant and 1 case of benign: five cases of previous progress (new lymph node enlargement and tumor marker values were significantly higher than before), two cases of empirical radiotherapy and chemotherapy were effective and one case of pneumoconiosis. The eight patients who were initially diagnosed as benign were finally diagnosed with three cases of tuberculosis, one case of sarcoidosis, two cases of organizing pneumonia, one case of allergic bronchopulmonary aspergillosis, and one case of community-acquired pneumonia (all improved after empirical treatment). There was no significant progress in the size and number of lymph nodes in 20 patients during the follow-up period. At the end of the follow-up period, the clinical diagnosis was reactive lymphadenopathy.

In summary, EBUS-TBNA provided a pathological confirmation for 566 patients (91.6%), of which 464 were malignant and 102 were benign. Details regarding the pathological diagnosis of EBUS-TBNA and clinical final diagnoses are presented in Table 2.

**Table 2:** Pathological diagnosis of EBUS-TBNA and clinical final diagnosis.

**Pathological diagnosis**	**Number of patients**	**Clinical final diagnosis**	**Number of patients**
**Malignant conditions**	**464**	**Malignant conditions**	**485**
Adenocarcinoma	135	Adenocarcinoma	150
Squamous cell carcinoma	113	Squamous cell carcinoma	118
Small cell lung cancer	96	Small cell lung cancer	97
Large cell lung cancer	2	Large cell lung cancer	2
Non-small cell lung cancer	5	Non-small cell lung cancer	4
Neuroendocrine tumor	9	Neuroendocrine tumor	9
Unclassified hypofractionated carcinoma	45	Unclassified hypofractionated carcinoma	43
unclassified malignancy	10	unclassified malignancy	9
Spindle cell carcinoma	1	Spindle cell carcinoma	1
Sarcomatoid carcinoma	2	Sarcomatoid carcinoma	2
Plasmacytoma/plasma cell carcinoma	2	Plasmacytoma/plasma cell carcinoma	2
Extrapulmonary metastatic cancer	28	Extrapulmonary metastatic cancer	30
Esophageal squamous cell carcinoma	3	Esophageal squamous cell carcinoma	3
Esophageal adenocarcinoma	1	Esophageal adenocarcinoma	1
Lymphoma	6	Lymphoma	7
adenoid cystic carcinoma	1	Adenoid cystic carcinoma	1
Neurogenic tumors	3	Neurogenic tumors	3
Perivascular epithelioid cell tumor	1	Perivascular epithelioid cell tumor	1
Malignant pleural mesothelioma	1	Malignant pleural mesothelioma	1
		Bronchial papilloma	1
**Benign lesions**	**82**	**Benign lesions**	**133**
Tuberculosis	31	Tuberculosis	40
Sarcoidosis	26	Sarcoidosis	28
Pneumoconiosis	2	Pneumoconiosis	4
Non-specific inflammation	18	Community-acquired pneumonia	20
Lymphoproliferative disorders	1	Organized pneumonia	6
Mediastinal cyst	2	Interstitial pneumonia	2
Mediastinal fibrosis	1	Bronchiectasis	2
Sinus histiocytosis	1	Cryptococcal infection	1
**Unclassified**	**72**	allergic bronchopulmonary aspergillosis	1
		Lymphoproliferative disorders	2
		Mediastinal cyst	2
		Mediastinal fibrosis	1
		Sinus histiocytosis	1
		Castleman's disease	1
		Thymic hyperplasia	1
		Inflammatory pseudotumor	1
		Reactive lymphadenopathy	20
**Total**	**618**	**Total**	**618**

7 cases of lymphoma including 2 non-Hodgkin lymphomas, 2 diffuse large B-cell lymphomas, 1 EBV-positive T-cell lymphoma, 1 mantle cell lymphoma, and 1 lymphoma unclassified. The primary tumors of 30 cases of extrapulmonary metastatic cancer included 5 breast cancer, 5 cervical cancer, 3 rectal cancer, 3 gastric cancer, 2 colon cancer, 2 esophageal cancer, 2 renal clear cell carcinoma, 1 tongue cancer, 1 endometrial cancer, 1 liver cancer, 1 tonsil cancer, 1 thyroid cancer, 1 thymus cancer, 1 prostate cancer, and 1 vaginal cancer.

### 3.3 Location and number of sampled lesions by EBUS-TBNA

A total of 999 targeted lesions were sampled, including mediastinal/hilar lymph nodes (*n* = 789), intrapulmonary masses (*n* = 193), mediastinal masses (*n* = 11), esophageal masses (*n* = 4), and tracheal masses (*n* = 2). The subcarinal node (level 7) was the most frequently punctured (*n* = 324, 32.4%), followed by the right lower paratracheal lymph node (level 4R) (*n* = 269, 26.9%), as indicated in Table 3.

**Table 3:** Characteristics of sampled lesions by EBUS-TBNA.

**Total lesions location**	***n =* 999**	**Positive rate**
Left pulmonary mass	69 (6.9%)	95.7%
Right pulmonary mass	124 (12.4%)	95.2%
Mediastinal masses	11 (1.1%)	100%
Peritracheal masses	2 (0.2%)	100%
Periesophageal masses	4 (0.4%)	100%
Subcarinal lymph node (7)	324 (32.4%)	89.5%
Right lower paratracheal lymph node (4R)	269 (26.9%)	91.4%
Left lower paratracheal lymph node (4L)	57 (5.7%)	89.5%
Right upper paratracheal lymph node (2R)	41 (4.1%)	95.1%
Right intrapulmonary lymph node (11R)	34 (3.4%)	85.3%
Left intrapulmonary lymph node (11L)	31 (3.1%)	77.4%
Right hilar lymph node (10R)	15 (1.5%)	80%
Right lobar lymph node(12R)	15 (1.5%)	86.7%
Left hilar lymph node (10L)	3 (0.3%)	100%
Number of sampled lymph nodes	789 (79.0%)	
Average number of samples	3 (1-8)	

### 3.4 Diagnostic performances of EBUS-TBNA

**Figure 2:**
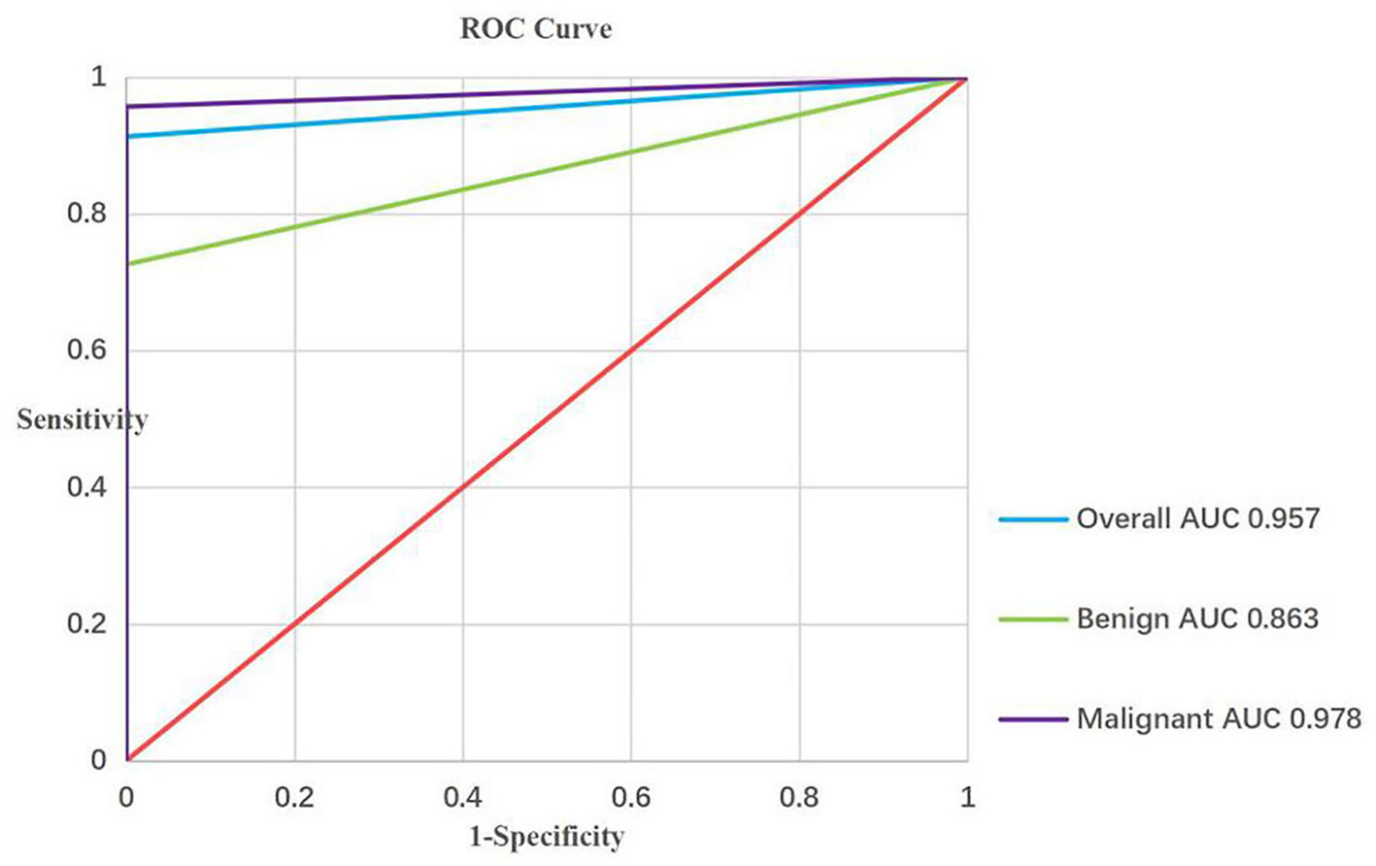
ROC curve analysis of the diagnostic performance of EBUS-TBNA for overall, malignant and bengin lesions. EBUS-TBNA, Endobronchial ultrasound-guided transbronchial needle aspiration.

Based on the results, the sensitivity, specificity, PPV, NPV, and accuracy of EBUS-TBNA for thoracic lesions were 91.3%, 100%, 100%, 27.8%, and 91.6% [95% confidence interval (CI): 89.1–93.6%], respectively. Diagnostic performance figures for EBUS-TBNA concerning total thoracic lesions, malignant lesions, and benign lesions are illustrated in Figure 2. Additionally, specific performance metrics for EBUS-TBNA when evaluating distinct diseases are detailed in Table 4.

**Table 4:** Diagnostic value of EBUS-TBNA in different diseases.

	**Sensitivity(%)**	**specificity(%)**	**PPV(%)**	**NPV(%)**	**Accuracy(%) (95% CI)**
**EBUS–TBNA**				
Overall *n =* 618	91.3	100	100	27.8	91.6 (0.891–0.936)
**Final diagnosis**				
Malignant lesions *n =* 485	95.7	100	100	48.8	95.8 (0.936–0.973)
Benign lesions *n =* 113	72.6	100	100	39.2	76.7 (0.684–0.834)
Primary Lung Cancer *n =* 437	96.1	100	100	54.1	96.3 (0.940–0.978)
Extrapulmonary metastatic cancer *n =* 30	93.3	100	100	90.9	96.0 (0.851–0.993)
Lymphoma *n =* 7	85.7	100	100	95.2	96.3 (0.791–0.998)
Tuberculosis *n =* 40	77.5	100	100	69.0	85.0 (0.729–0.925)
Sarcoidosis *n =* 28	92.9	100	100	90.9	95.8 (0.846–0.993)

### 3.5 The diagnostic performances of indications for EBUS-TBNA

The primary indication for EBUS-TBNA at our institution was the diagnosis and/or staging of thoracic space-occupying lesions in 482 patients (78.0%), followed by the diagnosis of isolated mediastinal and/or hilar lymphadenopathy (IMHL) in 69 patients (11.2%), and the diagnosis of suspected intrathoracic recurrence of previous malignancies in 67 patients (10.8%).

#### 3.5.1 Thoracic space-occupying lesions

Among the 482 patients (78.0%) diagnosed with thoracic space-occupying lesions, 775 lesions underwent biopsy, including 587 lymph nodes and 188 masses. In the 109 patients (22.6%) where only masses were biopsied, the diagnostic yield was 97.2%. For the 79 patients (16.4%) with biopsy of both lymph nodes and masses, three non-diagnostic results were observed, giving a diagnostic yield of 96.2% for this subgroup. Among the 294 patients (61.0%) who only experienced lymph node puncture, 31 patients had no diagnostic significance, with a diagnostic rate of 89.5%. In this group, six patients diagnosed with non-small cell lung cancer (NSCLC) after receiving chemoradiotherapy for mediastinal restaging had one case (16.7%) experiencing false negatives during follow-up.

Within this cohort, 410 patients (85.1%) received a diagnosis of malignant lesions encompassing various histopathological types, including adenocarcinoma (*n* = 135), squamous cell carcinoma (*n* = 104), small cell lung cancer (*n* = 93), (*n* = 1) large cell lung cancer, poorly differentiated carcinoma (*n* = 37), non-small cell lung cancer (*n* = 4), unclassified malignancies (*n* = 9), neuroendocrine carcinoma (*n* = 7), extrapulmonary metastatic carcinoma (*n* = 2), lymphoma (*n* = 5), esophageal squamous cell carcinoma (*n* = 3), and other malignancies (*n* = 10, 1 spindle cell carcinoma, 2 sarcomatoid carcinomas, 2 plasmacytoma/plasma cell carcinomas, 1 malignant pleural mesothelioma, 1 neurogenic tumor, 1 adenoid cystic carcinoma, 1 perivascular epithelioid cell tumor, and 1 bronchial papilloma). Additionally, there were 72 cases of benign lesions (14.9%), including cases of pneumonia (*n* = 24), tuberculosis (*n* = 24), sarcoidosis (*n* = 10), pneumoconiosis (*n* = 3), fungal infection (*n* = 2), organized pneumonia (*n* = 2), interstitial pneumonia (*n* = 2), bronchiectasis (*n* = 2), mediastinal cyst (*n* = 2), and mediastinal fibrosis (*n* = 1).

EBUS-TBNA accurately diagnosed 445 true positive results (including 395 cases of malignant lesions and 50 cases of benign lesions), with 37 false negative results. The sensitivity was 91.4%, the negative predictive value was 57.5%, and the diagnostic accuracy was 92.3% (95% CI: 89.5–94.5%).

#### 3.5.2 Isolated mediastinal and/or hilar lymphadenopathy

In the group of 69 patients (11.2%) with isolated mediastinal and/or hilar lymphadenopathy (IMHL), 128 lymph nodes were punctured, with the most common punctures occurring in level 7 groups (40.6%) and level 4R (35.2%). EBUS-TBNA successfully diagnosed malignancy in 20 out of 21 cases (95.2%). The pathological diagnoses included adenocarcinoma (*n* = 7), squamous cell carcinoma (*n* = 2), large cell lung cancer (*n* = 2), small cell lung cancer (*n* = 1), neuroendocrine tumor (*n* = 2), poorly differentiated carcinoma (*n* = 4), Epstein-Barr virus (EBV)-positive T-cell lymphoma (*n* = 1), and neurogenic tumors (*n* = 2). EBUS-TBNA revealed a benign diagnosis in 42 out of 48 cases (87.5%), including cases of sarcoidosis (*n* = 15), tuberculosis (*n* = 10), reactive lymphadenopathy (*n* = 14), pneumoconiosis (*n* = 1), lymphoproliferative disorder (*n* = 1), and sinus histiocytosis (*n* = 1). In this group, 7 (10.1%) false-negative cases (1 poorly differentiated carcinoma, 3 tuberculosis, 1 lymphoproliferative disorder, 1 Castleman's disease, and 1 inflammatory pseudotumor) were identified through other invasive procedures. The sensitivity, NPV, and accuracy were 87.3%, 66.7%, and 89.9% (95% CI: 79.6–95.5%), respectively.

#### 3.5.3 Suspected intrathoracic recurrence of previous malignancies

Among the 67 patients (10.8%), 41 cases (60.6%) were diagnosed with previous tumor recurrence, while 26 (39.4%) received a different diagnosis from their previous tumors. Out of these, 14 cases were identified as secondary primary tumors, and 12 cases were diagnosed as benign conditions. EBUS-TBNA definitively diagnosed 36 cases of recurrent malignant tumors, with the final pathological diagnosis corroborating their prior treated carcinomas through morphological and immunohistochemical analysis. Thirteen cases were determined to be secondary primary tumors. In 18 EBUS-TBNA cases, the results were initially regarded as non-malignant diagnoses, but further invasive procedures confirmed six of them as malignant tumors. The remaining 12 cases showed no further progression during the 6-month follow-up. The sensitivity, NPV, and accuracy were 89.1%, 66.7%, and 91.0% (95% CI: 80.9–96.3%), respectively.

### 3.6 Complications

During this study, only one patient (0.16%) experienced complications, specifically active bleeding during EBUS-TBNA procedures. The bleeding was successfully controlled after the local application of ice saline, adrenaline, and thrombin under the endoscope, as well as intravenous administration of hypophysin. No major complications, such as pneumothorax, mediastinal abscess, or significant bleeding, were observed.

## 4 Discussion

EBUS-TBNA is highly recommended as the first-line diagnostic and staging tool for lung cancer due to its excellent sensitivity, accuracy, and safety as per relevant guidelines (13, 14). Over the past decade, it has seen extensive use in diagnosing pulmonary and mediastinal lesions, encompassing various clinical conditions such as lung cancer, infectious diseases, sarcoidosis, and lymphoma. A summary of the diagnostic value of EBUS-TBNA in a series of recent an large-scale studies is presented in Table 5. In our study, the overall diagnostic accuracy, sensitivity, specificity, PPV and NPV were 91%, 88.3%, 100%, 100% and 89.2%, respectively (15–19). Notably, the negative predictive value appeared relatively lower overall, but it substantially improved when diseases were examined individually. This discrepancy may be attributed to our rigorous patient selection process and the absence of rapid on-site evaluation (ROSE).

**Table 5:** summary of the diagnostic value of EBUS-TBNA in a series of recent and large-scale studies.

	**Number of patients**	**Sensitivity (%)**	**Specificity (%)**	**PPV (%)**	**NPV (%)**	**Accuracy (%)**
Murthi et al. (15)	139	55.1	100	100	75.7	81.2
Bailey et al. (16)	327	89.7	100	100	85.1	89.9
Carbonari et al. (17)	52	86	100	100	77	90
Kosmas et al. (18)	234	88.3	100	100	89.2	91
Guarize et al. (19)	1891	91.7	100	100	78.5	93.6

Our study indicated a notable disparity in sensitivity and diagnostic rates between malignant and benign diseases, with significantly higher values for malignant cases (95.7% vs. 72.6% and 95.8% vs. 76.7%, respectively), especially for primary lung cancer. Furthermore, EBUS-TBNA provided ample samples, enabling immunohistochemical and molecular genotyping in 31.5% of cases, which proved pivotal in patient tumor typing and subsequent treatment strategies.

In regions like China and many developing countries where benign diseases such as sarcoidosis and tuberculosis are prevalent, EBUS-TBNA has proven effective in their diagnosis. The diagnostic accuracy of tuberculosis ranged between 78% and 92%, with a sensitivity of 69%-85% (20, 21), whereas sarcoidosis exhibited a diagnostic accuracy of 67%-94% and a sensitivity of 64%-96% (21–23). Our study demonstrated a tuberculosis diagnostic rate of 85.0% and a sensitivity of 77.5%, while for sarcoidosis, the diagnostic rate and sensitivity were 95.8% and 92.9%, respectively, in agreement with previous studies, highlighting its practical value.

We also analyzed the diagnostic performance of EBUS-TBNA in various chest clinical indications. In the diagnosis of thoracic space-occupying lesions, EBUS-TBNA demonstrated a sensitivity and diagnostic accuracy of 91.4% and 92.3%, respectively, consistent with previous findings (19). Notably, we observed a higher diagnostic positive rate in mass puncture (97.2%) compared to puncture mass and lymph node (96.2%) or lymph node only (89.5%). Additionally, EBUS-TBNA exhibited a 100% diagnostic positive rate when sampling masses near the mediastinum, trachea, and esophagus. Accordingly, we recommend combining lymph node puncture with diagnosis and staging wherever feasible alongside accessible nodules or masses to enhance the diagnostic positive rate. It is essential to remain vigilant regarding false negatives, especially in patients with NSCLC undergoing mediastinal restaging post-chemoradiotherapy. Our study indicated a 16.7% incidence of false negatives, potentially attributed to mediastinal necrosis and fibrosis induced by chemoradiotherapy, leading to a substantial number of false negative results (12, 24). Therefore, we recommend further surgical biopsies for pathological confirmation or close follow-up observation for patients with negative EBUS-TBNA pathological results to avoid treatment delays.

Moreover, our study revealed four newly diagnosed cases of primary esophageal cancer, indicating that EBUS-TBNA samples were sufficient for the pathological diagnosis of esophageal cancer. Even in severe cases of esophageal stenosis, EBUS-TBNA enabled specimen collection through fine needle puncture, enabling a more accurate staging by assessing the invasion of adjacent organs by esophageal tumors such as the tracheobronchial wall and mediastinal lymph nodes (25, 26).

EBUS-TBNA has played an important role in the diagnosis of IMHL as an alternative to mediastinoscopy. In a study, 120 patients with IMHL were evaluated and the sensitivity of EBUS-TBNA was determined to be 89.8% and the diagnostic rate 91.6% (22). In another prospective study, EBUS-TBNA yielded a sensitivity of 92% and a diagnostic rate of 92% (27). Our study corroborated these results, showing a sensitivity of 87.3% and a diagnostic accuracy of 89.9%. These findings suggest that EBUS-TBNA effectively reduces the need for subsequent surgical procedures. Notably, benign lesions in this group (69.6%) outweighed malignant lesions (30.4%), with sarcoidosis (31.2%) and tuberculosis (27.1%) being the most prevalent among benign lesions. A particularly interesting finding was the 100% sensitivity for sarcoidosis in this subgroup.

The sensitivity and diagnostic rates of EBUS-TBNA in suspected intrathoracic recurrence of previous malignancies were 89.1% and 91.0%, respectively. The absence of complications further emphasized its safety and efficacy as an alternative to other invasive procedures. Our study indicated that 61.2% of patients were diagnosed with tumor recurrence, with EBUS-TBNA confirming 87.8% of these cases, providing valuable guidance for the treatment of recurrent cases. Among those suspected of intrathoracic recurrence, 38.8% did not experience disease recurrence, unveiling a different diagnosis. Lung cancer (26.8%) emerged as the most recurrent tumor, with the longest follow-up period extending to 49 months.

EBUS-TBNA has been reported to have a low complication rate of around 0.15% (28) and is considered safe and well-tolerated, even in children and elderly patients (29, 30). Our study concurred with these findings, with only one patient experiencing active bleeding during EBUS-TBNA procedures, which was promptly resolved without observing any significant complications such as pneumothorax or mediastinal abscess. Additionally, we noted no surgery-related complications in elderly patients, further validating EBUS-TBNA's excellent safety profile.

Our study stands out due to its comprehensive analysis of EBUS-TBNA across different clinical indications and real-world settings, illustrating its usefulness and high accuracy. Nevertheless, the study is not without limitations. Being observational in nature, it is susceptible to confounding bias. The retrospective design indicates that the sample size was not pre-determined, and some variables (such as the number of aspirations, experience of endoscopists and pathologists, etc.) were not accounted for due to limited data from hospital records, potentially affecting EBUS-TBNA's diagnostic accuracy (31, 32). Furthermore, the lack of a standardized approach by a single respiratory physician in performing EBUS-TBNA might have introduced variations in surgical techniques. Additionally, only 50.0% (*n* = 36/72) of patients with negative EBUS-TBNA results underwent further invasive surgical confirmation, possibly leading to false negative results, although all patients underwent clinical and radiological follow-ups over 6 months to finalize the clinical diagnosis. Lastly, since the study was based on data collected from a single center, multicenter and randomized controlled trials with larger samples are required to validate these results and provide high-quality evidence.

## 5 Conclusion

In conclusion, clinical experience has demonstrated EBUS-TBNA to be a safe and efficacious minimally invasive diagnostic modality for thoracic lesions, warranting widespread clinical implementation. However, for suspected malignant cases with negative EBUS-TBNA pathology, comprehensive analysis is imperative, with additional diagnostic testing or clinical follow-up required to fully characterize the etiology of such lesions.

## Data availability statement

The original contributions presented in the study are included in the article/supplementary material, further inquiries can be directed to the corresponding authors.

## Ethics statement

The studies involving humans were approved by the Ethics Board of Zhongnan Hospital of Wuhan University (Approval number: 2023026K). The studies were conducted in accordance with the local legislation and institutional requirements. The ethics committee/institutional review board waived the requirement of written informed consent for participation from the participants or the participants' legal guardians/next of kin because the need for informed consent was waived due to the retrospective nature of the study.

## Author contributions

HL: Data curation, Investigation, Methodology, Writing—original draft. MZ: Data curation, Validation, Writing—original draft. RL: Data curation, Investigation, Writing—original draft. DW: Data curation, Methodology, Investigation, Writing—original draft. DX: Data curation, Funding acquisition, Project administration, Supervision, Writing—original draft, Writing—review & editing. YC: Data curation, Funding acquisition, Investigation, Project administration, Supervision, Writing—original draft, Writing—review & editing. ZC: Data curation, Formal analysis, Funding acquisition, Investigation, Methodology, Project administration, Supervision, Writing—original draft, Writing—review & editing.

## References

[1] Li Z Xu H Fan F. Approach to mediastinal fine needle aspiration cytology. Adv Anat Pathol. (2022) 29:337–48. 10.1097/PAP.0000000000000355 35838636

[2] Farsad M. FDG PET/CT in the staging of lung cancer. Curr Radiopharm. (2020) 13:195–203. 10.2174/1874471013666191223153755 31868151 PMC8206197

[3] Bonifazi M Tramacere I Zuccatosta L Mei F Sediari M Paonessa MC. Conventional versus ultrasound-guided transbronchial needle aspiration for the diagnosis of hilar/mediastinal lymph adenopathies: a randomized controlled trial. Respiration. (2017) 94:216–23. 10.1159/000475843 28531883

[4] Deng CJ Dai FQ Qian K Tan QY Wang RW Deng B. Clinical updates of approaches for biopsy of pulmonary lesions based on systematic review. BMC Pulm Med. (2018) 18:146. 10.1186/s12890-018-0713-6 30176840 PMC6122670

[5] Al-Ibraheem A Hirmas N Fanti S Paez D Abuhijla F Al-Rimawi D. Impact of ^(18)^F-FDG PET/CT, CT and EBUS/TBNA on preoperative mediastinal nodal staging of NSCLC. BMC Med Imaging. (2021) 21:49. 10.1186/s12880-021-00580-w 33731050 PMC7967993

[6] Steward M Dickson C. Sonography Endobronchial Assessment, Protocols, and Interpretation. StatPearls. Treasure Island, FL: StatPearls Publishing Copyright (2022). 34033340

[7] Divisi D Zaccagna G Barone M Gabriele F Crisci R. Endobronchial ultrasound-transbronchial needle aspiration (EBUS/TBNA): a diagnostic challenge for mediastinal lesions. Annals of translational medicine. (2018) 6:92. 10.21037/atm.2017.12.19 29666815 PMC5890040

[8] Ge X Guan W Han F Guo X Jin Z. Comparison of endobronchial ultrasound-guided fine needle aspiration and video-assisted mediastinoscopy for mediastinal staging of lung cancer. Lung. (2015) 193:757–66. 10.1007/s00408-015-9761-3 26186887

[9] Chen YB Jiang JH Mao JY Huang JA. Diagnostic value of endobronchial ultrasound-guided transbronchial needle aspiration (EBUS-TBNA) in solitary mediastinal, hilar lymphadenectasis, or peribronchial lesions: six cases reports and review of literature. Medicine. (2016) 95:e5249. 10.1097/MD.0000000000005249 27858883 PMC5591131

[10] Yang J De Cardenas J Nobari M Miller R Cheng G. Narrative review of tools for endoscopic ultrasound-guided biopsy of mediastinal nodes. Mediastinum. (2020) 4:34. 10.21037/med-20-25 35118302 PMC8794368

[11] Labarca G Folch E Jantz M Mehta HJ Majid A Fernandez-Bussy S. Adequacy of samples obtained by endobronchial ultrasound with transbronchial needle aspiration for molecular analysis in patients with non-small cell lung cancer. Systematic review and meta-analysis. Ann Am Thorac Soc. (2018) 15:1205–16. 10.1513/AnnalsATS.201801-045OC 30011388

[12] Muriana P Rossetti F. The role of EBUS-TBNA in lung cancer restaging and mutation analysis. Mediastinum. (2020) 4:23. 10.21037/med-20-24 35118291 PMC8794354

[13] Vilmann P Clementsen PF Colella S Siemsen M De Leyn P Dumonceau JM. Combined endobronchial and esophageal endosonography for the diagnosis and staging of lung cancer: European society of gastrointestinal endoscopy (ESGE) guideline, in cooperation with the european respiratory society (ERS) and the European society of thoracic surgeons (ESTS). Eur J Cardio-Thoracic Surg Off. (2015) 48:1–15. 10.1093/ejcts/ezv194 26034060

[14] Silvestri GA Gonzalez AV Jantz MA Margolis ML Gould MK Tanoue LT. Methods for staging non-small cell lung cancer: diagnosis and management of lung cancer, 3rd ed: American college of chest physicians evidence-based clinical practice guidelines. Chest. (2013) 143:e211S–e50S. 10.1378/chest.12-2355 23649440

[15] Murthi M Donna E Arias S Villamizar NR Nguyen DM Holt GE. Diagnostic accuracy of endobronchial ultrasound-guided transbronchial needle aspiration (EBUS-TBNA) in real life. Front Med. (2020) 7:118. 10.3389/fmed.2020.00118 32318581 PMC7154097

[16] Bailey N Krisnadi Z Kaur R Mulrennan S Phillips M Slavova-Azmanova N. pragmatic application of endobronchial ultrasound-guided transbronchial needle aspiration: a single institution experience. BMC Pulm Med. (2019) 19:155. 10.1186/s12890-019-0909-4 31429741 PMC6701134

[17] Carbonari A Rossini L Marioni F Camunha M Saieg M Bernardi F. Value of endobronchial ultrasound-guided transbronchial needle aspiration (EBUS-TBNA) in the diagnosis of lung and mediastinal lesions. Revista da Assoc Med Brasileira. (1992). 66:1210–6. 10.1590/1806-9282.66.9.1210 33027447

[18] Kosmas K Kosmas A Riga D Kyritsis C Riga NG Tsiambas E. Impact of endobronchial ultrasound-guided transbronchial needle aspiration (EBUS-TBNA) on lung carcinoma staging: a retrospective study. Cureus. (2021) 13:e17963. 10.7759/cureus.17963 34660150 PMC8516022

[19] Guarize J Casiraghi M Donghi S Diotti C Vanoni N Romano R. Endobronchial ultrasound transbronchial needle aspiration in thoracic diseases: much more than mediastinal staging. Can Res J. (2018) 2018:4269798. 10.1155/2018/4269798 29686741 PMC5857308

[20] Ortakoylu MG Iliaz S Bahadir A Aslan A Iliaz R Ozgul MA. Diagnostic value of endobronchial ultrasound-guided transbronchial needle aspiration in various lung diseases. J Bras Pneumol Pub Oficial Soc Bras Tisil. (2015) 41:410–4. 10.1590/S1806-37132015000004493 26578131 PMC4635086

[21] Low SY Koh MS Ong TH Phua GC Anantham D. Use of endobronchial ultrasound-guided transbronchial needle aspiration (EBUS-TBNA) in the diagnosis of granulomatous mediastinal lymphadenopathy. Ann Acad Med Singapore. (2014) 43:250–4. 10.47102/annals-acadmedsg.V43N5p250 24919489

[22] Temiz D In E Kuluöztürk M Kırkıl G Artaş G Turgut T. The role of endobronchial ultrasound-guided transbronchial needle aspiration in the differential diagnosis of isolated mediastinal and/or hilar lymphadenopathy. Diagn Cytopathol. (2021) 49:1012–21. 10.1002/dc.24807 34078002

[23] Cetinkaya E Ozgül MA Tutar N Ozgül G Cam E Bilaçeroglu S. The diagnostic utility of real-time EBUS-TBNA for hilar and mediastinal lymph nodes in conventional TBNA negative patients. Ann Thorac Cardiovasc Surg. (2014) 20:106–12. 10.5761/atcs.oa.12.02072 23411844

[24] Cetinkaya E Usluer O Yilmaz A Tutar N Çam E Özgül MA. Is endobronchial ultrasound-guided transbronchial needle aspiration an effective diagnostic procedure in restaging of non-small cell lung cancer patients? Endoscopic Ultrasound. (2017) 6:162–7. 10.4103/eus.eus_3_17 28621292 PMC5488518

[25] Chi J Lian SS Yang Q Luo GY Xu GL. The utility of EBUS-TBNA in the diagnosis of suspected intrathoracic recurrence after esophageal cancer surgery. Jpn J Clin Oncol. (2020) 50:602–8. 10.1093/jjco/hyz212 31943047

[26] Zuccatosta L Mei F Bonifazi M Gasparini S. Historical eye: from traditional to endobronchial ultrasound-guided needle aspiration and beyond. Curr Opin Pulm Med. (2023) 29:3–10. 10.1097/MCP.0000000000000924 36474461

[27] Navani N Lawrence DR Kolvekar S Hayward M McAsey D Kocjan G. Endobronchial ultrasound-guided transbronchial needle aspiration prevents mediastinoscopies in the diagnosis of isolated mediastinal lymphadenopathy: a prospective trial. Am J Respir Crit Care Med. (2012) 186:255–60. 10.1164/rccm.201203-0393OC 22652031 PMC3423452

[28] Gu P Zhao YZ Jiang LY Zhang W Xin Y Han BH. Endobronchial ultrasound-guided transbronchial needle aspiration for staging of lung cancer: a systematic review and meta-analysis. Eur J Cancer. (2009) 45:1389–96. 10.1016/j.ejca.2008.11.043 19124238

[29] Madan K Iyer H Madan NK Mittal S Tiwari P Hadda V. Efficacy and safety of EBUS-TBNA and EUS-B-FNA in children: a systematic review and meta-analysis. Pediatr Pulmonol. (2021) 56:23–33. 10.1002/ppul.25124 33073498

[30] Niwa H Oki M Ishii Y Torii A Yamada A Shinohara Y. Safety and efficacy of endobronchial ultrasound-guided transbronchial needle aspiration (EBUS-TBNA) for patients aged 80 years and older. Thoracic cancer. (2022) 13:1783–7. 10.1111/1759-7714.14454 35523730 PMC9200877

[31] Mohan A Naik S Pandey RM Mills J Munavvar M. Diagnostic utility of endobronchial ultrasound guided transbronchial needle aspiration for mediastinal lesions: a prospective three year, single centre analysis. Thorac Cancer. (2011) 2:183–9. 10.1111/j.1759-7714.2011.00063.x 27755856

[32] Flandes J Giraldo-Cadavid LF Perez-Warnisher MT Gimenez A Fernandez-Navamuel I Alfayate J. Learning curves and association of pathologist's performance with the diagnostic accuracy of linear endobronchial ultrasound transbronchial needle aspiration (EBUS-TBNA): a cohort study in a tertiary care reference centre. BMJ Open. (2022) 12:e051257. 10.1136/bmjopen-2021-051257 36261243 PMC9582308

